# The PAR network: redundancy and robustness in a symmetry-breaking system

**DOI:** 10.1098/rstb.2013.0010

**Published:** 2013-11-05

**Authors:** Fumio Motegi, Geraldine Seydoux

**Affiliations:** 1 Temasek Lifesciences Laboratory, National University of Singapore, 1 Research Link, Singapore 117604, Republic of Singapore; 2 Mechanobiology Institute, National University of Singapore, 1 Research Link, Singapore 117604, Republic of Singapore; 3 Department of Biological Sciences, National University of Singapore, 1 Research Link, Singapore 117604, Republic of Singapore; 4 Department of Molecular Biology and Genetics and HHMI, Johns Hopkins University School of Medicine, Baltimore MD 21205, USA

**Keywords:** polarity, PAR proteins, Cdc42, robustness, feedback loops, modelling of biological networks

## Abstract

To become polarized, cells must first ‘break symmetry’. Symmetry breaking is the process by which an unpolarized, symmetric cell develops a singularity, often at the cell periphery, that is used to develop a polarity axis. The *Caenorhabditis elegans* zygote breaks symmetry under the influence of the sperm-donated centrosome, which causes the PAR polarity regulators to sort into distinct anterior and posterior cortical domains. Modelling analyses have shown that cortical flows induced by the centrosome combined with antagonism between anterior and posterior PARs (mutual exclusion) are sufficient, in principle, to break symmetry, provided that anterior and posterior PAR activities are precisely balanced. Experimental evidence indicates, however, that the system is surprisingly robust to changes in cortical flows, mutual exclusion and PAR balance. We suggest that this robustness derives from redundant symmetry-breaking inputs that engage two positive feedback loops mediated by the anterior and posterior PAR proteins. In particular, the PAR-2 feedback loop stabilizes the polarized state by creating a domain where posterior PARs are immune to exclusion by anterior PARs. The two feedback loops in the PAR network share characteristics with the two feedback loops in the Cdc42 polarization network of *Saccharomyces cerevisiae*.

## 1. Introduction

Polarization of the *Caenorhabditis elegans* zygote has been a focus of study for over 25 years. Genetic, molecular and cell biological analyses have identified the key cytoskeletal elements and regulators that pattern the cell cortex. These analyses are beginning to reveal the architecture of the polarity network that initiates, amplifies and maintains polarity in the zygote. In §2 of this review, we describe the modules that make up the polarity network. In §3, we summarize *in vivo* and *in silico* experiments that identify redundancies among the modules and suggest possible sources of robustness in the network. Finally, in §4, we highlight common themes that emerge from comparing the architecture of the *C. elegans* network to that of *Saccharomyces cerevisiae*.

## 2. The PAR network

### (a) Overview of the polarization process

**Figure 1.**
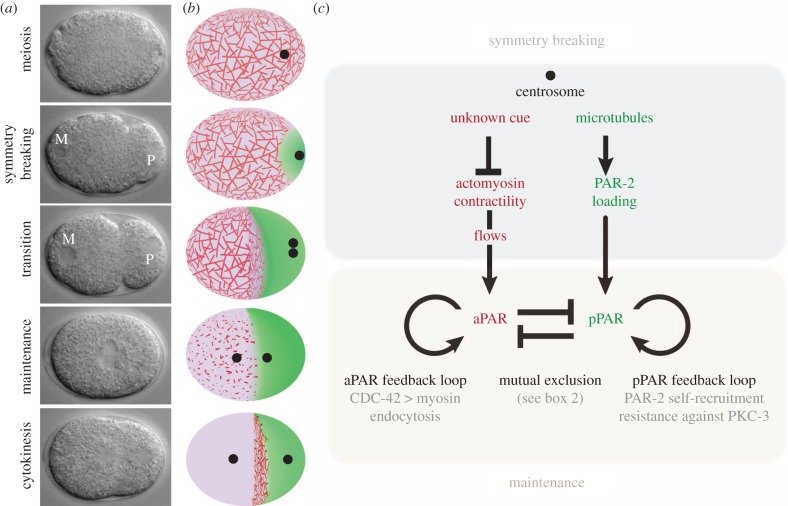
Overview of polarization. (*a*) Nomarski photomicrographs of a wild-type *C. elegans* zygote undergoing polarization. The series covers 14 min. M and P label the maternal and paternal pronuclei, which fuse in the maintenance phase. Note the transient furrow (pseudocleavage) that forms at the transition between the symmetry-breaking and maintenance phases. (*b*) Diagrams depicting a wild-type *C. elegans* zygote undergoing polarization. Cortical accumulation of aPARs and pPARs are depicted in pink and green, respectively. The sperm centrosome, which separates after symmetry breaking, is depicted as a black dot. Actomyosin cables and puncta are depicted in red. Note that the actomyosin network switches from a cable network during symmetry breaking to a punctate network during maintenance, and back to a cable network during cytokinesis. (*c*) Schematic showing the architecture of the PAR polarity network. Two redundant symmetry-breaking inputs feed into the PAR network, which consists of a mutual exclusion module and two positive feedback loops.

The *C. elegans* one-cell zygote divides asymmetrically to generate two daughter blastomeres with different fates: a larger somatic blastomere in the anterior, and a smaller germline blastomere in the posterior. The anterior/posterior axis is generated by a process that begins before mitosis under the influence of the paternally provided centrosome (figure 1). At fertilization, the *C. elegans* zygote is unpolarized and contains two pronuclei typically located at opposite ends of the zygote: the maternal pronucleus, which undergoes two rounds of meiotic divisions using an acentriolar spindle, and the paternal pronucleus and its associated centrosome. After meiosis, the centrosome begins to nucleate microtubules and approaches the cortex; the position of the centrosome at that time defines the posterior side of the embryo (symmetry breaking) [1]. Eventually, the paternal pronucleus and centrosome leave the cortex to meet the maternal pronucleus in the cytoplasm. The pronuclei fuse and establish a centrally located mitotic spindle. During anaphase, the spindle becomes displaced towards the posterior. As a consequence of the eccentric location of the spindle, cytokinesis splits the zygote into two unequally sized cells.

**Figure 2.**
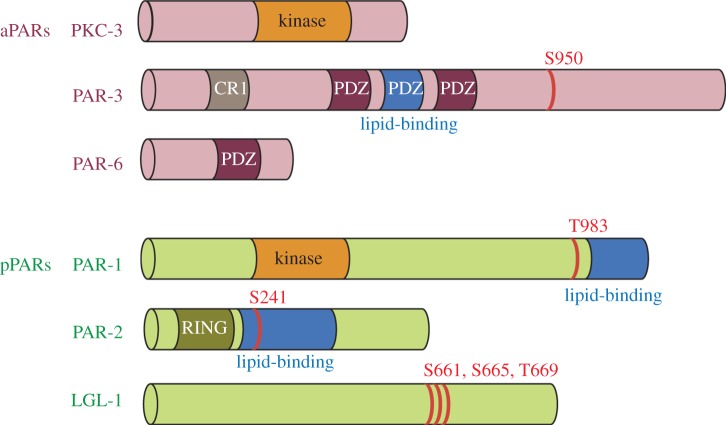
Anterior and posterior PAR proteins. Phosphorylation sites are depicted in red. PKC-3 phosphorylates pPARs, and PAR-1 phosphorylates PAR-3.

The first molecular insights into polarization came from the identification of mutants that cause the zygote to divide symmetrically [2,3]. The *par* (PARtitioning defective) mutants define six loci, *par-1–6*. Two additional loci, the atypical protein kinase C (aPKC) homologue *pkc-3*, and the *lethal giant larvae* homologue *lgl-1*, were identified later as genetic and physical interactors [4–6]. With the exception of *par-2*, all the *par* genes have direct homologues in other animals that have been implicated in the polarization of many different cell types [7–9]. The PAR proteins can be divided into three groups based on their localizations in the polarized zygote. PAR-4 and PAR-5 remain uniformly distributed at the cortex throughout the polarization process [10,11]. The posterior PARs (pPARs: PAR-1, PAR-2 and LGL-1) localize to half the cortex on the centrosome side [4,5,12,13] and the anterior PARs (aPARs: PAR-3, PAR-6 and PKC-3) localize to the other half [6,14,15] (figure 2). In all cases, localization to the cortex is not absolute, and each PAR protein can also be detected in the cytoplasm. Fluorescent recovery after photobleaching (FRAP) and fluorescent correlation spectroscopy (FCS) analyses have revealed that GFP::PAR-2 and GFP::PAR-6 readily exchange between the cortex and the cytoplasm and also diffuse laterally along the cortex [16–18]. How the PAR proteins associate with the cortex is not completely understood, but PAR-1, PAR-2 and PAR-3 all have domains predicted to interact directly with phospholipids (box 1), suggesting that these proteins contact the inner leaflet of the plasma membrane. Remarkably, even after polarization, there appears to be no diffusion barrier between the anterior and posterior domains: GFP::PAR-2 diffuses into the anterior domain and GFP::PAR-6 diffuses in the posterior domain [16]. Extensive mixing is prevented by reciprocal inhibitory interactions that lower the affinity of pPARs for the cortex occupied by aPARs and vice versa (mutual exclusion; box 2).

Box 1. **How do PAR proteins localize to the cortex?**

Drugs that disrupt actin microfilaments prevent the localization of PAR-3 and PAR-2 to the cell cortex even before and during symmetry breaking [12,19], suggesting that at least some of the PARs interact with the actomyosin network that forms under the plasma membrane. Interactions with lipids, however, have also been implicated in PAR localization, raising the possibility that the PARs also interact directly with the plasma membrane.

PAR-1 and PAR-2 both contain domains that can bind acidic phospholipids *in vitro. In vivo*, localization of PAR-2 to the cell periphery depends on a central domain in PAR-2 rich in basic amino acids, raising the possibility that PAR-2 uses electrostatic interactions to interact with phospholipids at the plasma membrane [20,21]. The C-terminal domain of PAR-1 (kinase-associated 1 (KA1) domain) contains a structurally-conserved binding site for acidic phospholipids [22]. In the yeast PAR-1 homologue Kcc4p, the KA1 domain is required but not sufficient for membrane localization *in vivo*; binding to a membrane-associated protein partner is also required. *C. elegans* PAR-1 binds to PAR-2 [20], and PAR-2 is required for localization of PAR-1 to the cortex [12], suggesting that PAR-2 could serve as the membrane protein partner for PAR-1. When PAR-3 is depleted, some PAR-1 can localize to the membrane without PAR-2 [12]. PAR-1 may therefore interact with the membrane in two ways: a weak interaction dependent on the KA1 domain, and a stronger interaction involving PAR-2. The latter is required for PAR-1 to resist exclusion by aPARs (box 2).

Localization of PAR-3 to the cortex also appears to depend both on binding to lipids and on binding to other proteins, including PAR-3 itself. PAR-3 contains at least two domains required for robust localization to the cell periphery: CR1 and PDZ2 [23]. The CR1 domain of PAR-3 promotes oligomerization of *Drosophila* and mammalian PAR-3 [24,25], and is required for *C. elegans* PAR-3 to interact with itself in a yeast two-hybrid assay [26,27]. Mammalian and *Drosophila* PAR-3 utilizes the PDZ2 domain and the C-terminal part for binding to phospholipids [28,29]. In *C. elegans* PAR-3, the PDZ2 domain is required for maximum localization to the cortex, and is also essential for recruiting PAR-6 and aPKC-3 there [23].

PAR-6 and PKC-3 require PAR-3 for maximal localization to the cortex, but there is also evidence that PAR-6 and PKC-3 can associate with the cortex in a PAR-3-independent manner. PAR-6 and PKC-3 localize to many puncta on the cortex, including 65% that contain PAR-3 and 35% that do not [30]. Localization to PAR-3-negative puncta depend on the Rho GTPase CDC-42 [30], which interacts directly with PAR-6 and plasma membrane lipids [31–34]. Additionally, in zygotes depleted for the Hsp90 co-chaperone CDC-37, PAR-6 no longer requires PAR-3 for cortical association but still requires CDC-42 [30]. Thus, multiple interactions contribute to the cortical association of the anterior PAR complex.

Box 2. **What mechanisms underlie mutual exclusion?**

Exclusion of pPARs from the anterior cortex depends on phosphorylation by PKC-3. PAR-1, PAR-2 and LGL-1 can each be phosphorylated *in vitro* by the mammalian homologue of PKC-3, aPKC. In the case of PAR-2, phosphorylation by aPKC has been shown to directly reduce affinity for phospholipids, perhaps because the negatively charged phosphates interfere with electrostatic interactions between the basic domain of PAR-2 and acidic phospholipids [20,21]. Consistent with this hypothesis, S → E and S → A substitutions at predicted PKC phosphorylation sites in PAR-2 have opposite effects on PAR-2 localization *in vivo*: S → E substitutions reduce PAR-2 localization to the cortex and S → A substitutions cause PAR-2 to localize throughout the cortex. Similar results have been obtained for LGL-1 and PAR-1 [5,20], suggesting that all three pPAR proteins are direct targets of PKC-3.

Exclusion of aPARs from the posterior cortex depends on the combined action of PAR-1 and LGL-1. Overexpression of PAR-2 can displace PAR-3 from the cortex, but only if PAR-1 is also present, suggesting that PAR-2 cannot displace aPARs on it own [21]. As first shown in *Drosophila*, PAR-1 phosphorylates PAR-3 on a conserved site in the C-terminal domain [35]. This site is not essential in wild-type zygotes [23], but is required to exclude PAR-3 from the posterior domain of zygotes that have been prevented from undergoing cortical flows [20]. LGL-1 also is not essential, but its overexpression can rescue loss of PAR-2 [5]. How LGL-1 excludes anterior PARs is not completely understood. Immunoprecipitation studies have suggested that binding of LGL-1 to PAR-6 could interfere with association of the aPAR complex with the cortex [5]. LGL-1 has also been shown to lower PAR-6 levels [36] and to suppress myosin recruitment to the posterior cortex, which could free up more myosin for the aPAR feedback loop [4].

Mutual exclusion of aPARs and pPARs also requires PAR-4 and PAR-5, the two PAR proteins that remain uniformly distributed at the cortex during polarization. PAR-4 is homologous to the serine–threonine kinase LKB1, which phosphorylates and activates PAR-1 in *Drosophila* and mammals [37]. PAR-5 is a 14-3-3 protein predicted to bind to sites phosphorylated by PAR-1 [35]. PAR-4 and PAR-5 therefore could participate in mutual exclusion by promoting exclusion of PAR-3 by PAR-1. The phenotypes of *par-4* and *par-5* zygotes, however, are much more severe than *par-1* zygotes, so these proteins are likely to also participate in other aspects of the polarization process [11,38,39].

### (b) Symmetry breaking

Immediately before symmetry breaking, aPARs are uniformly distributed throughout the cortex and pPARs are predominantly in the cytoplasm. During symmetry breaking, aPARs clear from the cortex nearest the centrosome, and pPARs accumulate there (figure 1*b*). This reorganization is the combined result of two independent mechanisms operating in parallel during symmetry breaking: cortical flows and cortical loading of PAR-2 (figure 1*c*).

#### (i) Cortical flows

At the completion of the meiotic divisions, a network of interconnected actomyosin foci and cables assembles under the plasma membrane, causing the membrane to ruffle as cables randomly contract and release [40]. When the paternally provided centrosome approaches the cortex, the cable network dissipates at the point of contact, and the rest of the network flows towards the opposite pole. The onset of cortical flows coincides temporally and spatially with disappearance of GFP::ECT-2 from the cortex nearest the centrosome [41]. ECT-2 is a guanine-exchange factor (GEF) that activates the small GTPase RHO-1 to assemble the contractile network [41–43]. A local reduction in ECT-2/RHO-1 activity could create the anterior/posterior gradient in contractility that drives flow [44], but the mechanism that inactivates ECT-2/RHO-1 near the centrosome is not yet known.

Coincident with the onset of cortical flows, aPARs disappear from the cortex nearest the centrosome and become enriched in the anterior (figure 1*b*). Live imaging revealed that GFP::PAR-6 foci on the cortex move away from the centrosome with the same speed and direction as myosin foci [40], suggesting that cortical flows ‘carry’ the anterior PAR complex towards the anterior. Consistent with this model, *in vivo* measurements of flow rates and GFP::PAR-6 residence time at the cortex support the view that the PARs are mobilized by passive advective transport, as would be molecules embedded in a thin layer of fluid above a flowing cortex [16,45].

#### (ii) PAR-2 loading

Because of the antagonism between aPARs and pPARs (box 2), displacement of aPARs by cortical flows promotes loading of pPARs on the posterior cortex. pPARs, however, can also access the posterior cortex in the absence of cortical flows [20,46,47]. In zygotes lacking the myosin regulatory light chain MLC-4, the actomyosin cable network does not form, and actin and myosin remain uniformly distributed at the cortex with no flows. Under these conditions, aPAR and pPAR domains still form, although more slowly [20,46,47]. Genetic analyses indicate that PAR-2 loads first and recruits PAR-1, and PAR-1, in turn, phosphorylates PAR-3, leading to exclusion of aPARs from the PAR-2/PAR-1 domain [12,20].

Before symmetry breaking, PAR-2 is kept off the cortex by phosphorylation by PKC-3 [21] (box 2). How does the centrosome allow PAR-2 to overcome this exclusion? The timing and position of PAR-2 loading correlates with microtubule nucleation by the centrosome [48–50]. Treatments that delay microtubule nucleation delay PAR-2 loading both in wild-type and *mlc-4(RNAi*) zygotes [20,49,51]. Consistent with a direct role for microtubules, PAR-2 binds microtubules with moderate affinity *in vitro*, and localizes transiently to the microtubule-rich halo that forms around the centrosome immediately before symmetry breaking. Two PAR-2 mutants selected not to bind microtubules *in vitro* do not localize to centrosome microtubules and are not able to break symmetry in *mlc-4(RNAi*) embryos. *In vitro* experiments suggest that microtubules compete with PKC-3 for access to PAR-2. In the absence of microtubules, aPKC readily phosphorylates PAR-2 and prevents PAR-2 from binding to phospholipids. Addition of microtubules to the kinase reaction blocks phosphorylation of PAR-2 and restores binding to phospholipids [20]. Together, these findings suggest that microtubules nucleated by the centrosome directly protect PAR-2 from PKC-3, allowing unphosphorylated PAR-2 to access the membrane nearest the centrosome.

### (c) Maintenance of PAR domains

After symmetry breaking, the paternal pronucleus/centrosome complex moves back towards the centre of the zygote to meet the maternal pronucleus and initiate the first mitotic division. In mitotic prophase, the actomyosin cable network in the anterior cortex disassembles and is replaced with a finer punctate network that maintains a higher concentration of actin and myosin [40]. The aPAR/pPAR boundary is at the cell midpoint and is maintained there by a complex set of interactions among the PAR proteins. First, mutual exclusion between aPARs and pPARs prevents extensive mixing between the domains (box 2). Second, PAR activities in each domain provide self-reinforcing positive inputs that maintain the domains. We refer to these activities as the aPAR and pPAR feedback loops.

#### (i) aPAR feedback loop

**Figure 3.**
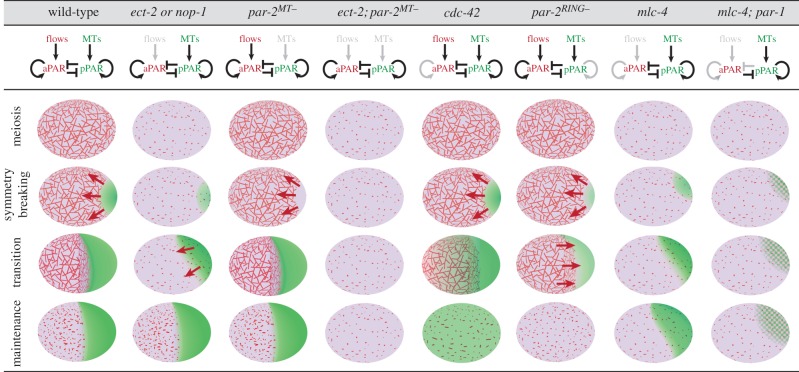
Phenotypes of mutants in the polarization network. Genotypes are indicated at the top of the figure. The schematic below depicts the PAR network as in figure 1, where flows and MTs refer to the two centrosome-dependent, symmetry-breaking inputs. Grey shading highlights the modules disrupted in the mutants. The embryo diagrams are as described in figure 1. Cortical accumulation of aPARs and pPARs are depicted in pink and green, respectively. The checked pattern of pink and green in the last series is meant to represent overlap between aPARs and PAR-2. Cortical flows are depicted with arrows; note the ‘late’ aPAR-dependent flows in the *ect-2*/*nop-1* and *par-2^RING−^* mutants.

During the maintenance phase, localization of aPARs to the cortex becomes dependent on the small GTPase CDC-42. PAR-6 binds directly to CDC-42 and is required for the enrichment of GFP::CDC-42(Q61L) (active form of CDC-42) in the anterior domain. Zygotes that express a PAR-6 mutant that cannot bind CDC-42 lose PAR-6 from the cortex during the maintenance phase [31]. At that same time, PAR-2 expands throughout the cortex, presumably because PKC-3 is lost along with PAR-6. Similar results are seen when CDC-42 is inactivated by RNAi (figure 3). *cdc-42(RNAi*) zygotes also exhibit reduced levels of myosin enrichment in the anterior cortex during the maintenance phase [36,41]. Loss of cortical myosin is also observed in zygotes depleted for PAR-3 or the CDC-42-associated kinase MRCK-1 [40,52]. Together, these findings indicate that enrichment of myosin and aPARs in the anterior cortex switches from a RHO-1-dependent mechanism to an aPAR/CDC-42-dependent mechanism during maintenance.

Endocytic recycling has also been implicated in the maintenance of PAR-6 at the cortex. The dynamin homologue DYN-1 stimulates endocytosis in the anterior domain. During the maintenance phase, GFP::PAR-6 puncta are occasionally seen near the anterior membrane colocalized with endocytic markers, suggesting that cortical PAR-6 is internalized and quickly recycled [53,54]. Like depletion of CDC-42, depletion of DYN-1 reduces PAR-6 levels at the cortex and expands the PAR-2 domain during the maintenance phase. Why endocytic recycling is required to maintain high levels of aPAR activity in the anterior cortex is not known.

#### (ii) pPAR feedback loop

Maintenance of the pPAR domain depends on PAR-2. In *par-2* mutants, aPARs become enriched to the anterior cortex by cortical flows during symmetry breaking, but neither PAR-1 nor LGL-1 [5,12] are recruited to the posterior cortex, and the aPARs re-enter the posterior cortex during the maintenance phase [55]. The return of aPARs is associated with posterior-directed flows that re-equilibrate myosin throughout the cortex [40]. How PAR-2 normally prevents these flows is not fully understood, but may be linked, at least in part, to PAR-2's ability to recruit PAR-1 [20,21]. PAR-1 and LGL-1 both contribute to exclusion of aPARs [4,5,20,36,55]; by contrast, PAR-2 cannot exclude aPARs on its own [21] (box 2).

Interestingly, formation of a stable PAR-2 domain does not require exclusion of anterior PARs. For example, in *par-1(RNAi*) zygotes, GFP::PAR-6 returns to the posterior cortex during the maintenance phase [55], but GFP::PAR-2 remains on the posterior cortex [21]. Similarly, in *mlc-4(RNAi*) zygotes that express a kinase-dead form of PAR-1 or a non-phosphorylatable form of PAR-3, PAR-3 and PKC-3 remain uniformly distributed at the cortex, but PAR-2 and kinase-dead PAR-1 still form a posterior domain near the centrosome [20]. These observations indicate that once on the cortex, PAR-2 can resist exclusion by PKC-3. As described above, PAR-2 initially gains access to the cortex by interacting with centrosomal microtubules, which protect PAR-2 from PKC-3. Mutant PAR-2 that cannot bind microtubules cannot access the cortex on its own, but can do so if wild-type PAR-2 is also present [20]. One possibility is that cortical PAR-2 can recruit PAR-2 molecules from the cytoplasm directly. Such self-recruitment may be what allows PAR-2 to expand beyond the site of centrosome/cortex contact [20]. Expansion of the PAR-2 domain requires the RING domain of PAR-2: a PAR-2^RING^ mutant localizes to the centrosome, but cannot spread to the cortex beyond [20,21]. FRAP analyses suggest that the RING domain increases the residence time of PAR-2 at the cortex [20]. FCS experiments support the view that PAR-2 assembles into slow-diffusing complexes when on the cortex [18]. Together, these observations suggest that slow-diffusing PAR-2 at the membrane creates a ‘PKC-3-immune domain’ able to recruit additional PAR-2 and PAR-1 molecules from the cytoplasm. Creation of a PKC-3 immune domain allows PAR-2, PAR-1 (and possibly LGL-1) to associate with the posterior cortex even in the presence of PKC-3.

## 3. Redundancy and robustness

As summarized above, symmetry breaking and maintenance involve several mechanisms that function in parallel. How redundant are these mechanisms and how do they contribute to the overall robustness of the system? This question can be answered by examining the phenotypes of mutants that disrupt specific mechanisms or ‘modules’ in the PAR network (figure 3).

### (a) The two symmetry-breaking mechanisms are mostly redundant

In wild-type embryos, PAR-2 first appears on the cortex approximately 600 s before cytokinesis. Mutations in PAR-2 that eliminate microtubule-binding (PAR-2^MT−^) delay PAR-2 loading by 30 s, presumably the time required for cortical flows to remove PKC-3 [20]. Mutations that eliminate centrosome-induced cortical flows (e.g. *nop-1* mutants, see below) do not delay PAR-2 loading, but delay expansion of the PAR-2 domain by approximately 150 s [47,56]. Despite the delays, most PAR-2^MT−^ and *nop-1* zygotes still fully polarize before cytokinesis and are viable. This ‘rescue’ is largely due to two important properties of the system: (i) either symmetry-breaking mechanism is sufficient to engage the aPAR and pPAR feedback loops; and (ii) the feedback loops are able to amplify and ‘lock in’ even small, transient changes in PAR distribution (discussed further below). Thus, although the two symmetry-breaking mechanisms make detectable contributions at the cellular level, they are mostly redundant at the organismal level.

What are the centrosome cue(s) that trigger symmetry breaking? At the time of symmetry breaking, the centrosome is ‘maturing’: recruiting pericentriolar proteins, such as the Aurora-A kinase AIR-1, Cyclin E-Cdk2, SPD-2 and SPD-5, and nucleating microtubules. Mutations that interfere with maturation also interfere with polarization [48,50,57–61]. In zygotes depleted of tubulin by RNAi, symmetry breaking is delayed and coincides with the formation of a smaller, late-forming aster at the centrosome [49]. Depletion of tubulin also blocks polarization by the meiotic spindle, a microtubule-rich acentriolar structure that is also able to polarize zygotes [48]. As described above, microtubules bind to PAR-2 directly and promote its loading on the cortex. Microtubules are therefore one cue used by the centrosome to break symmetry, but whether microtubules are the only cue is not known. In particular, whether microtubules are responsible for cortical flows has not yet been demonstrated. Pericentriolar proteins could also play a role, but it has been difficult to separate a specific requirement for these factors from a general requirement for microtubule assembly. One study also proposed a role for CYK-4, a sperm-enriched Rho GTP activating protein (GAP) [42]; however, a CYK-4 mutant lacking GAP activity does not have a polarity phenotype [56]. During cytokinesis, RhoA activity is regulated by a combination of positive signals from the central spindle (CYK-4) and negative signals from the centrosomes/astral microtubules (aster). The aster-pathway requires NOP-1 and is sufficient to induce a transient furrow between the asters [56]. Remarkably, NOP-1 is also required for centrosome-induced flows and for the formation of a transient furrow during symmetry breaking (pseudocleavage) [56,62–64]. The parallels between aster-directed furrowing and pseudocleavage suggest that the same mechanisms are used to inactivate RHO-1 near the asters during cytokinesis and symmetry breaking.

The actin regulator Arp2/3 has also been implicated in symmetry breaking. Arp2/3 nucleates a small population of F-actin in the posterior cortex [51]. Loss of Arp2/3 reduces the strength of cortical flows, delays microtubule nucleation by the centrosome, and interferes with PAR-2 loading. Actin polymerization, therefore, could contribute to both symmetry-breaking pathways, but the mechanisms involved are not yet known.

The possibility that the centrosome use multiple, redundant cues to break symmetry may explain why symmetry breaking is so robust even under conditions where centrosome function is compromised. For example, in zygotes depleted of gamma tubulin by RNAi, movement of the centrosome towards the cortex is delayed. In these zygotes, the centrosome is still deep in the cytoplasm when changes in actomyosin dynamics are first detected on the posterior cortex, and the centrosome moves towards the cortex after cortical and cytoplasmic flows have started [65]. These observations suggest that the centrosome can affect cortical dynamics at a distance, and that cortical and cytoplasmic flows promote recruitment of the centrosome to the cortex. Centrosome/cortex proximity, in turn, promotes the formation of asymmetric PAR domains [57,66–68]. Mutants that cause the centrosome to leave the cortex prematurely do not polarize the PARs; inactivation of dynein, which forces the centrosome to remain near the cortex, rescues polarity in these mutants [66,68]. Together, these observations suggest that the centrosome uses both long-range and short-range mechanisms to break symmetry. The long-range cues are not yet known, but may be related to the aster-dependent cues that inactivate RHO-1 at the cell poles during cytokinesis and create cortical flows. The short-range cues may include PAR-2 bound to microtubules and possibly other cues that reinforce/sustain cortical flows.

### (b) The aPAR feedback loop can amplify changes in PAR distribution

Under wild-type conditions, the symmetry-breaking inputs from the centrosome are sufficient to generate full-size PAR domains and, consequently, the PAR feedback loops are required primarily to maintain polarity. Under conditions where symmetry breaking is inefficient, however, the aPAR feedback loop can also participate in symmetry breaking by helping to contract/expand the aPAR/pPAR domains. This is best seen in *ect-2* or *nop-1* mutants. These mutants lack centrosome-induced cortical flows and only form a small pPAR domain during symmetry breaking (figure 3). Later during mitosis, the pPAR domain rapidly expands to half the cortex [47,49]. Expansion coincides with activation of ‘late’ cortical flows that restrict myosin and aPARs to the anterior. As mentioned above, during mitosis, myosin regulation switches from RHO-1 to aPAR/CDC-42. The aPAR/CDC-42 loop is responsible for the ‘late’ flows: depletion of *par* or *cdc-42* activity blocks all flows in *rho-1*, *ect-2* and *nop-1* zygotes [47,52,56]. These observations indicate there are, in fact, two types of cortical flows capable of mobilizing the aPARs: early RHO-1-dependent flows that form during symmetry breaking, and late CDC-42-dependent flows that can be triggered during mitosis. Either type is able to concentrate the aPARs to the anterior half of the zygote. Only the RHO-1-dependent flows are able to break symmetry, because RHO-1 activity is sensitive to the position of the centrosome. CDC-42-dependent flows, in contrast, cannot break symmetry on their own. Anterior-directed CDC-42-dependent flows are only seen in zygotes that lack centrosome-induced cortical flows but that are still able to load PAR-2 on the posterior cortex owing to its microtubule-binding affinity. Presumably, PAR-2/PAR-1 loading creates a local reduction in aPAR/CDC-42 activity near the centrosome, which activates anterior-directed flows [47]. The late flows are required to expand the pPAR domain to a wild-type full-size (approx. 50% of the length of the zygote): in zygotes with no early or late flows, the pPAR domain remains small (approx. 30% egg length) [20]. A likely possibility is that the late flows promote growth of the pPAR domain by displacing PKC-3 from the posterior cortex. A reduction in PKC-3 could lower the concentration of PAR-2 required in the posterior domain to neutralize PKC-3 and recruit more PAR-2, allowing PAR-2 to spread over an even larger domain.

Cortical flows also contribute to the proper orientation of PAR domains. Because the centrosome contacts the cortex at a random position, the pPAR domain often initially forms off the long axis of the egg and is repositioned later. This repositioning correlates with cortical flows: repositioning occurs during symmetry breaking in wild-type zygotes, during the late flows in *nop-1* and *ect-2* zygotes, and does not occur in *mlc-4(RNAi*) zygotes, which lack all flows [47,56]. One possibility is that the oval shape of the zygote forces the flows to align with the long axis by creating anisotropies in cortical tension [44].

### (c) The pPAR feedback loop locks in the polarized state

As described above, the pPAR feedback loop is required to stabilize polarity after symmetry breaking. This function requires PAR-2 activity, and, in particular, the RING domain of PAR-2. A PAR-2^RING^ mutant loads on the posterior cortex during centrosome-induced cortical flows, but is displaced by returning aPARs during the maintenance phase. Interestingly, this defect is partially alleviated when the PKC-3 phosphorylation sites in the PAR-2^RING^ mutant are also mutated [21]. Similarly, the lethality of *par-2(0*) mutants can be partially rescued by lowering *par-6* levels or increasing LGL-1 levels [4,5,15,69]. These genetic interactions suggest that PAR-2's primary role is to neutralize excess aPAR activity. As described above, PAR-2 cannot displace aPARs from the posterior cortex on its own, but is able to form a ‘PKC-3-immune domain’ that can recruit additional PAR-2 and PAR-1 molecules from the cytoplasm even in the continued presence of PKC-3 [20]. We suggest that this ability, combined with slow diffusion of cortical PAR-2, ‘locks in’ the polarized state by permanently transforming the posterior cortex into a PKC-3-resistant zone. How PAR-2 is able to neutralize PKC-3 without displacing PKC-3 from the cortex is not understood.

In summary, efficient polarization depends on two mostly redundant symmetry-breaking mechanisms that activate two non-redundant positive feedback loops: one required to convert the transient symmetry-breaking inputs into a lasting change in the posterior cortex (pPAR loop), and one required to expand and/or maintain asymmetry throughout the cell (aPAR loop). Mutual exclusion between aPARs and pPARs (box 2) prevents mixing between the anterior and posterior domains but is not sufficient in the absence of the PAR feedback loops to stabilize or amplify PAR domains (figure 3).

### (d) Modelling the PAR network

Now that many of the players and interactions have been defined in the polarization system, mathematical modelling is becoming an increasingly powerful tool to build and test possible polarization mechanisms [70]. Reaction–diffusion models, in which patterns are generated by interactions between proteins with different diffusion rates [71], have been developed to explore how interactions amongst the PAR proteins could generate bistability (two stable states: unpolarized and polarized). In these models, the PAR proteins are considered as two complexes, aPAR and pPAR, that are either associated with the cortex or present in the cytoplasm. Relative levels on cortex and in cytoplasm are a function of the rate of lateral diffusion along the cortex, the rate of exchange between cortex and rapidly diffusing cytoplasmic pools, and cortical dissociation caused by mutual exclusion [45,72,73].

The model of Tostevin & Howard [73] is based on the hypothesis that aPARs have a higher affinity for a contractile cortex. The model incorporates feedback between the aPAR proteins and the actomyosin network, by proposing that aPARs promote flows by reducing resistance in the cortex, whereas pPARs reduce flows by increasing resistance. The model was the first to provide a complete description of the polarization process, but does not account for the observation since then that actomyosin asymmetry is not essential for symmetry breaking. The possibility that aPARs and pPARs have different affinities for different cortices (contractile or non-contractile) could, however, contribute to the amplification of PAR domains and/or polarity maintenance.

The model of Dawes and Munro introduced the concept of nonlinearity in mutual exclusion as a mechanism to stabilize PAR domains during the maintenance phase, without the need for an asymmetric actomyosin cortex [72]. A simple linear negative feedback (where one aPAR complex can displace one pPAR complex and vice versa) would be too sensitive to minor fluctuations in PAR levels and would require too perfect a balance of reaction kinetics to achieve bistability. In any region of the cortex, if one PAR type gained a slight advantage due to stochastic fluctuations, then this advantage would be reinforced until that PAR domain spread to the entire cortex. The primary requirement for bistability is that mutual exclusion be nonlinear (more than one aPAR complex is required to displace one pPAR complex or vice versa). In their model, Dawes and Munro introduced nonlinearity by postulating the existence of a dimeric form of PAR-3 with increased affinity for the cortex. Consistent with that hypothesis, as described above, PAR-3 has an oligomerization domain that is required *in vivo* to stabilize the aPAR domain (box 1).

Goehring *et al*. [45] combined mutual exclusion with an advection model to also describe the role of cortical flows during symmetry breaking. The advection model treats the actomyosin cortex as a thin contractile layer that can entrain the cytoplasm around it to create fluid flows under the plasma membrane [44]. The model uses parameters of lateral diffusion on the membrane and exchange between membrane and cytoplasm determined experimentally by FRAP analysis for GFP::PAR-2 and GFP::PAR-6 [16]. Comparison of flow speed and of PAR lifetime at the membrane suggests that the two occur on similar enough time scales to mobilize or ‘advect’ the PARs towards the anterior. At the aPAR/pPAR boundary, lateral diffusion of one complex is countered by cortical exclusion by the opposing complex. As in the Dawes and Munro model, nonlinearity in the mutual exclusion module imparts bistability to the system, allowing it to switch from a single anterior PAR domain to two mutually exclusive PAR domains upon application of flows.

The models based on mutual exclusion require that aPAR and pPAR levels be balanced to prevent runaway expansion of the pPAR domain and maintain the boundary at the midpoint of the cell. To accommodate this requirement, Goehring *et al*. [45] proposed that the PAR proteins are recruited from finite cytoplasmic pools, which become depleted during symmetry breaking causing expansion of the pPAR domain to ‘stall out’. The model predicts that small (20–30%) changes in aPAR or pPAR levels should shift the boundary and larger changes should prevent polarization altogether. Overexpression of PAR-2 does shift the boundary towards the anterior, but the shift is more modest than predicted [45]. In addition, mutants that reduce PAR-6 levels by 40% or have an over 25% increase in PAR-6 level are still able to polarize normally [36,74]. Another prediction of the cytoplasmic pool requirement is that, for a given aPAR/pPAR ratio, the aPAR/pPAR boundary should reach the same position regardless of the symmetry-breaking input. The boundary, however, is not positioned properly in embryos that lack centrosome-induced flows, and can be repositioned during mitosis [47,56,75]. Finally, none of the models account for the observation that PAR-2 can form a stable cortical domain under conditions where aPARs remain symmetric (e.g. zygotes lacking PAR-1 kinase activity) [20,21].

The limitations of the models are consistent with the experimental evidence, summarized above, that mutual exclusion is not sufficient to support robust polarization *in vivo*; the aPAR and pPAR feedback loops are also required. Hypersensitivity to PAR balance as predicted by the models has been observed experimentally, but in embryos that lack centrosome-induced flows, the aPAR loop or the pPAR loop [47,49,69]. We suggest that redundancy of symmetry-breaking inputs and the PAR feedback loops buffer the network from imprecisions in the aPAR/pPAR balance. Consistent with this view, a recent study comparing synthetic polarity networks also concluded that positive feedback loops increase robustness in networks based on mutual inhibition [76]. Another oversimplification in the models so far has been to assume that aPARs and pPARs behave as single, constant species that compete only by cortical exclusion. In fact, PAR proteins within each group are likely to make distinct contributions to the polarization process. For example, PAR-2 can neutralize the effect of PKC-3 on the posterior cortex without displacing aPARs [20]. This and many other activities specific to each PAR protein will have to be considered to build more realistic models.

### (e) Unsolved mystery: positioning the boundary

PAR domain dynamics stop when the aPAR/pPAR boundary reaches the middle of the zygote. How is that position determined? As mentioned above, modelling analyses have shown that boundary placement is determined, in principle, by the balance of aPAR and pPAR activities [45,72]. Experimental evidence shows that the intensity of cortical contractions also plays a role. The redundant RhoGAPs RGA-3 and RGA-4 limit RHO-1-dependent contractility: *rga-3/4(RNAi*) zygotes assemble a more dense actomyosin cable network which prolongs the contraction, resulting in a smaller aPAR domain and a larger pPAR domain during symmetry breaking [77,78]. RHO-1-dependent hyper-contractility is also seen in zygotes depleted of the TAO kinase KIN-18 [79]. Interestingly, KIN-18 binds to PAR-3 in the yeast two-hybrid assay. *par-3* mutants show weak contractility during symmetry breaking, and this phenotype is suppressed by loss of *kin-18* [79]. Thus, careful regulation of RHO-1 activity, which may involve feedback regulation from PAR-3, is critical to calibrate the size of PAR domains during symmetry breaking.

PAR-1 is also required to limit expansion of the pPAR domain. *par-1* mutants have exaggerated cortical flows and form a larger pPAR domain during symmetry breaking. Loss of the PAR-1 kinase substrates MEX-5 and MEX-6 suppresses this phenotype and reduces the size of the pPAR domain [55]. MEX-5 and MEX-6 are redundant CCCH zinc finger proteins that relocalize from the posterior cytoplasm to the anterior cytoplasm, as PAR-1 becomes enriched in the posterior [80,81]. One possibility is that polarization of MEX-5/6 functions as a negative feedback loop: when MEX-5/6 levels fall below a threshold in the posterior cytoplasm in response to increased PAR-1 asymmetry, expansion of the posterior domain stops. How MEX-5/6, two RNA-binding proteins in the cytoplasm, affects PAR dynamics on the cortex is not known.

Finally, there is also a mechanism during mitosis that refines the position of the aPAR/pPAR boundary to align it with the cytokinesis furrow. For example, the anteriorly displaced aPAR/pPAR boundary in *rga-3/4* zygotes is repositioned during cytokinesis to match the cytokinetic furrow [75]. As mentioned above, the actomyosin network that assembles during cytokinesis resembles the one formed during symmetry breaking, except that it is focused in the middle of the cell. As in symmetry breaking, the PAR boundary follows actomyosin flows into the cytokinetic furrow. Recent observations indicate that PAR-3 and PAR-6 are required partially redundantly for successful cytokinesis and may play a role in spatial organization of the cytokinetic furrow [82].

Another fascinating aspect of the PAR network is the ability to self-correct. Wild-type embryos occasionally develop two pPAR domains, one near the centrosome and one near the meiotic spindle remnant (also a microtubule-rich structure) [12,49]. As the domain near the centrosome expands, the pPAR domain near the meiotic spindle resorbs. Mutations that delay centrosome maturation and reduce the strength of cortical flows allow the meiotic pPAR domain to be maintained for longer [60,83]. These observations suggest that the system is able to respond to competing cues and ‘choose’ one, perhaps because of competition for limiting reagents.

## 4. Common themes and future prospects

A comparison of the *C. elegans* PAR network with the network that mediate polarization of budding yeast highlights some common themes and interesting differences. *Saccharomyces cerevisiae* uses Cdc42 to polarize, but does not have a PAR network. Symmetry breaking begins with the stochastic or induced formation of a concentrated focus of active, GTP-bound Cdc42 at the membrane. As in *C. elegans*, the initial symmetry-breaking event is amplified by two positive feedback loops [84].

The first feedback loop involves an interplay between short-range auto-activation and long-range inhibition of Cdc42. Slow-diffusing GTP-Cdc42 on the membrane recruits a fast-diffusing activator from the cytoplasm. The activator converts nearby Cdc42 molecules to the active GTP-bound state, and these, in turn, recruit more activator (short-range activation). Depletion of the activator from the cytoplasm prevents Cdc42 activation at other membrane sites (long-range inhibition). This loop is similar to the PAR-2 feedback loop where ‘RING active’ slow-diffusing PAR-2 at the membrane can recruit fast-diffusing PAR-2 from the cytoplasm, allowing the PAR-2 domain to grow. Growth eventually stalls when the cytoplasmic pool of PAR-2 is depleted [45]. Long-range inhibition in the PAR-2 loop could be the mechanism that eliminates the second pPAR domain that forms near the meiotic spindle remnant in some zygotes [12,49].

The second feedback loop in the yeast system involves Cdc42-dependent activation of actin-dependent processes (transport and vesicular recycling) that increase delivery of Cdc42 to the symmetry-breaking site. This loop is reminiscent of the aPAR feedback loop, which uses cortical flows and endocytic recycling to concentrate aPARs in the anterior cortex. Interestingly, in yeast, the actin feedback loop is considered a secondary, slower response [26]. By contrast, in the much larger *C. elegans* zygote, cortical flows contribute significantly to the speed and efficiency of the polarization process. It is tempting to speculate that animal cells evolved new ways to use mechanical forces to propagate symmetry-breaking inputs over larger distances [85].

To build even more realistic and comprehensive models of the PAR network, it will be important to measure parameters (i.e. rates of PAR diffusion, membrane binding, and mutual exclusion) during the different phases of polarization. Changes in actomyosin in the transition from symmetry breaking to maintenance could alter PAR diffusion and/or sensitivity to mutual exclusion. Further biochemical and genetic studies are also required to fully define the partners and biochemical properties of PAR proteins [63,64]. Super high-resolution imaging techniques may also help refine our understanding of where the PAR proteins are relative to each other, the membrane and the cortex. Ultimately, the relative simplicity of the *C. elegans* zygote promises that reiterated cycles of modelling and experimental explorations will eventually lead to a complete, system-level understanding of the polarization process in an animal cell.
